# Editorial: Immune Cell Lineage Reprogramming in Cancer

**DOI:** 10.3389/fimmu.2021.838464

**Published:** 2022-01-19

**Authors:** Jianmei W. Leavenworth, Lewis Zhichang Shi, Xi Wang, Haiming Wei

**Affiliations:** ^1^ Department of Neurosurgery, University of Alabama at Birmingham, Birmingham, AL, United States; ^2^ The O’Neal Comprehensive Cancer Center, University of Alabama at Birmingham, Birmingham, AL, United States; ^3^ Department of Microbiology, University of Alabama at Birmingham, Birmingham, AL, United States; ^4^ Department of Radiation Oncology, University of Alabama at Birmingham, Birmingham, AL, United States; ^5^ Institute of Infectious Diseases, Beijing Key Laboratory of Emerging Infectious Diseases, Beijing Ditan Hospital, Capital Medical University, Beijing, China; ^6^ Beijing Institute of Infectious Diseases, Beijing, China; ^7^ National Center for Infectious Diseases, Beijing Ditan Hospital, Capital Medical University, Beijing, China; ^8^ Department of Oncology, Capital Medical University, Beijing, China; ^9^ Hefei National Laboratory for Physical Sciences at Microscale, Division of Life Science and Medicine, University of Science and Technology of China, Heifei, China; ^10^ Institute of Immunology, University of Science and Technology of China, Heifei, China

**Keywords:** tumor immunity, regulatory T-cells, helper T-cells, tumor-associated macrophages, natural killer cells, epigenetic regulation, lineage reprogramming, cancer immunotherapy

Cancer immune evasion, as a result of prominent immunosuppression, is a major barrier to effective anti-tumor immunity and immunotherapy. Both adaptive and innate immune cells in cancer have shown phenotypic and functional instability by reprogramming into different cell subsets or states that impact tumor growth, progression or metastasis. Our Research Topic has attracted 18 contributions from 145 authors, which collectively cast a largely complete picture of our current understanding of the immune cell reprogramming and associated mechanisms in cancer, with or without therapeutic interventions.

## Reprogramming Adaptive Immune Cells in Cancer

As one of the major anti-tumor cytotoxic T lymphocytes (CTLs), CD8^+^ T-cells generally reside in the tumor with exhausted and dysfunctional states (1). CD8^+^ T-cell exhaustion is a contentious topic in the field of cancer research, as two models are proposed to explain this formation: one, the attrition of effector cells upon chronic antigen stimulation, and two, early bifurcation of an exhausted lineage in tumorigenesis (1, 2). Using two distinct T-cell receptor (TCR) transgenic and transplantable tumor models, Sullivan et al. demonstrate that although both tumor-specific and tumor-nonspecific bystander CD8^+^ T-cells traffic to solid tumors *via* the chemokine receptor CXCR3, the former cells are exhausted, while the latter cells within the same tumor microenvironment (TME) retain memory and functional activity, which supports the notion that chronic TCR stimulation is the central driver of T-cell exhaustion. In contrast, Busselaar et al. provide a new perspective that the early priming without CD4^+^ T-cell help differentiates CD8^+^ T-cells into a predysfunctional state to express the transcription factor TCF-1 and coinhibitory receptors, such as PD-1 (3). Subsequent antigen stimulation drives their differentiation into TCF-1^−^ terminally exhausted cells dependent on the transcription factor TOX (4, 5). Importantly, PD-1 blockade along with CD27 costimulation and other alternative approaches that recapitulate CD4^+^ T-cell help could fully rescue the predysfunctional state, suggesting new strategies for cancer immunotherapy. Interestingly, memory bystander CD8^+^ T-cells reported by Sullivan et al. do not express high levels of PD-1. It is not clear if these cells respond to PD-1 blockade as efficiently as predysfunctional CD8^+^ T-cells. Nevertheless, these studies highlight the plasticity of intratumoral CD8^+^ T-cells that could be exploited for cancer immunotherapy.

CD4^+^ T-cells not only provide help to CD8^+^ T-cells to optimize CTL response, but also directly regulate the magnitude and quality of anti-tumor immunity (6). In addition, emerging studies have demonstrated that CD4^+^ T-cells provide help to B-cells to induce anti-tumor humoral antibody response and the formation of tumoral tertiary lymphoid structures, which serve as predictive and prognostic factors in patients with cancer and those receiving immunotherapies (7, 8). Conversely, accumulation of CD4^+^ regulatory T-cells (Tregs) in many tumors is a hallmark of immunosuppressive TME (9). The versatility of CD4^+^ T-cell functional activity lies at heterogenous subsets and states of these cells, as reviewed by DiToro and Basu, who also provide a comprehensive review of the complex transcriptional networks and dynamic responses of CD4^+^ T-cell subsets in intestinal inflammation and colorectal cancer. Additionally, they address therapeutic targeting *via* CD4^+^ T-cell functional plasticity, including manipulation of the colonic microbiota. In a study conducted by Fraga et al., some patients with oral squamous cell carcinoma (OSCC) have increased tumor-infiltrating T helper (T_H_)2-like and CCR8^+^ effector T-cells (Teff) and Tregs, which are subsets associated with poor prognosis. Co-culture assays and proteomic analysis of the secretome from OSCC have further identified an important link with increased production of prostaglandin E2 and activated vitamin D signaling to the T_H_2-like Treg and Teff phenotype and induction of CCR8 but inhibition of cytokine secretion in Teff. Moreover, malignant OSCC samples express elevated CCL18, the CCR8 ligand, to promote CCR8 upregulation in Teff, forming an immunosuppressive feedback loop. A more focused review of Tregs is provided by Dixon et al., who have discussed the stability and suppressive function of tumoral Tregs, including a subset of effector Tregs, follicular regulatory T (T_FR_) cells that are implicated in the regulation of anti-tumor humoral response (10), and the therapeutic potential by targeting Treg reprogramming for cancer treatments.

## Reprogramming Innate Immune Cells in Cancer

In addition to the adaptive immune system, components of innate immune system contribute to tumor growth, progression and response to immunotherapy. There are diverse types of innate immune cells. Some display tumor-killing capacity, while others exhibit pro-tumoral property. Natural killer (NK) cells by virtue of their natural cytotoxicity are crucial in the control of various types of cancer. Hu et al. provide an overview of how the TME alters NK cell phenotype, function, metabolism and migration, while Xia et al. focus on the epigenetic regulation of NK cell heterogeneity in cancer, and discuss epi-drugs used to target NK-mediated anti-tumor immunity. Like suppressive lymphocytes, innate myeloid cells, including myeloid-derived suppressor cells (MDSC) and tumor-associated macrophages (TAMs), also accumulate in many types of tumors. Several transcription factors, such as C/EBPβ and c-Rel, are reported to regulate MDSC differentiation and function (11, 12), but the lineage-specific regulator remains unclear. Fultang et al. propose a c-Rel-C/EBPβ enhanceosome containing these known transcription factors in myeloid precursors as a unified mechanism for the regulation of MDSC signature genes during their differentiation in response to aberrant inflammatory cytokine signals, suggesting potential therapeutic strategies *via* specifically targeting MDSC. A detailed review of TAMs is presented by both Ricketts et al. and Pan et al., who have discussed the TAM plasticity and approaches targeting TAMs to improve the anti-tumor response. The former has also presented interesting proactive questions by pointing out that the *in vitro* M1/M2 experimental model cannot accurately represent the intra-tumoral TAM heterogeneity, while new technologies, such as single-cell RNA-sequencing and spatial localization, would help refine our understanding of TAMs. Although this collection cannot provide an exhausted list of innate immune cells, the above studies highlight the importance of innate regulation of tumor immunity, and the potential to harness the plasticity of these innate immune cells for cancer therapy.

## Reprogramming the Tumor Microenvironment

Cancer is increasingly viewed as a “tumor ecosystem” in which tumor cells interact with other tumor cells, stromal cells and all kinds of immune cells to constitute an immunosuppressive TME that is a major obstacle to effective anti-cancer immunity. Instead of focusing on a specific type of immune cells, Yang and Wang have discussed the epigenetic regulation of tumor cells, intratumoral immune cells, tumor-immune crosstalk and the heterogeneity of TME from a systemic view, proposing that combined epi-drugs and immunotherapy is an effective strategy for cancer therapy. This review has also briefly presented how microbiota-derived signals or metabolites could epigenetically regulate the TME, an open area for future exploration. The TME creates a condition that is disadvantageous to the nutrient uptake and metabolism of immune effector cells. Li Y et al. have discussed how TME-derived metabolites reprogram immune cells *via* epigenetic regulation, supporting a strategy to enhance the efficacy of immunotherapy using metabolic modifiers. An overview of the ovarian cancer TME by Luo et al. has also described tumor-infiltrating immune cells that are modulated by genetic and epigenetic factors, particularly noncoding RNAs, intrinsically or extrinsically from tumor cells. The cytokine signaling and components like JAK-STATs that mediate tumor-immune interactions in the TME are also a focus of this review. The complexity and plasticity of TME is impacted by the genomic heterogeneity of tumor cells, which can be assessed *via* targeted next-generation sequencing. Using this technology, Lin et al. are able to define the spatial heterogeneity of multiple tumors of resected multifocal hepatocellular carcinoma. Moreover, circulating-free DNA from matched preoperative peripheral blood effectively captures these genomic alterations, serving as a promising tool to inform cancer progression and to potentially guide the selection of best treatments, including immunotherapies, for cancer patients.

## Reprogramming Immune Cells and TME in Response to Cancer Therapy

Cancer therapies that are aimed to converting the TME from immunosuppressive (cold) to immune-supportive (hot) are expected to induce the immune cell lineage reprogramming, which is potentially targetable for new therapeutic interventions due to its reversibility. Various cancer immunotherapeutic approaches are currently being employed in the clinic of which immune checkpoint inhibitors (ICIs) targeting PD-1, PD-L1 and CTLA4 have shown the most promising results, despite that the overall response rates remain at low levels in many types of cancer, especially for those cancers with high levels of immunosuppressive cells in the TME or insufficient infiltration of effector cells into tumor. Based on this potential mechanistic link, combined treatments with ICIs and angiogenesis inhibitors that can reduce immunosuppression but enhance effector cell infiltration into tumor to reprogram the TME could improve the outcome of ICI-based therapy (Ren et al.). This review has also summarized the preclinical and clinical studies of using the combined approach for the treatment of advanced non-small cell lung cancer, in addition to a detailed discussion of the mechanisms of vascular endothelial growth factor signaling in tumor immune evasion and progression. In contrast to the beneficial effects, immune-related adverse effects are one of the major concerns for ICI-based therapy. Kim et al. report that IFNγ^+^IL-17^−^ CD8^+^ T and CXCR3^+^CCR6^+^ T_H_17/T_H_1 cells were enriched and clonally expanded in the bronchoalveolar lavage fluid from 11 patients with acute myeloid leukemia and myelodysplastic syndrome after ICI-based therapy, suggesting that these cells may contribute to ICI-related pulmonary complications and serve as predictive and diagnostic biomarkers for these adverse effects.

It is interesting that the involvement of immune regulation is also identified in the standard-of-care treatments like surgical resection and chemotherapy. Shibuya et al. identified a tissue-repair-promoting Ym1^+^Ly6C^hi^ monocyte subset that results from the inflammation post-resection of primary tumor and promotes lung metastasis of circulating tumor cells at least partly *via* expressing metalloproteinase-9 and CXCR4. These findings suggest this specific immunomodulatory monocyte subset as a predictive biomarker for metastatic recurrence after primary tumor resection. It is known that cisplatin chemotherapy is widely used in multiple tumors, but it produces severe side effects including neurotoxicity and immunosuppression. A safe and effective complementary treatment is required to prevent toxicity and preserve bone marrow hematopoiesis and peripheral immune responses. Li S et al. revealed that electroacupuncture can induce PAC1-mediated neuromodulation of hematopoiesis and alleviate immunosuppression in naïve and tumor-bearing mice during cisplatin treatments. This study may open an interesting research avenue in which the neuro-immune axis can be manipulated for the treatment of cancer and therapy-related side effects.

## Conclusions

This Research Topic “*Immune Cell Lineage Reprogramming in Cancer*” provides updates on the influences of immune cell lineage reprogramming on tumor initiation, progression, and outcomes of therapy. Although cancer immunotherapy has emerged as a promising modality for cancer patients, much remains to be learned given the importance of TME regulation that is complicated by the plasticity and heterogeneity of immune cells and tumor cells. We (the editors) strongly believe that each article published under this Research Topic will help in the discovery of new cellular and molecular candidates or pathways for the development of strategies against cancer.

## Author Contributions

JWL initiated and organized the Research Topic. All authors made substantial, direct and intellectual contributions to the work, and approved it for publication.

## Funding

JWL is supported by the University of Alabama at Birmingham (UAB) faculty start-up funds, DoD W81XWH-18-1-0315 and NIH R01AI148711. XW is supported by grants from the Ministry of Science and Technology of People’s Republic of China (2014CB910100) and the National Natural Science Foundation of China (81972652). LZS has received research fund from the V Foundation for Cancer Research (V2018-023), American Cancer Society Institutional Research Grant (91-022-19), Varian (a Siemens Healthineers company), NIH R21CA230475, R21CA259721-A1 and Start-up fund from the Department of Radiation Oncology at UAB.

## Conflict of Interest

LZS received financial support from Varian, a Siemens Healthineers company.

The remaining authors declare that the research was conducted in the absence of any commercial or financial relationships that could be construed as a potential conflict of interest.

## Publisher’s Note

All claims expressed in this article are solely those of the authors and do not necessarily represent those of their affiliated organizations, or those of the publisher, the editors and the reviewers. Any product that may be evaluated in this article, or claim that may be made by its manufacturer, is not guaranteed or endorsed by the publisher.
